# Intersectoral and Pro-Equity Approaches in Health Policy

**DOI:** 10.5334/aogh.4863

**Published:** 2025-09-08

**Authors:** Fabiana C. Saddi, Stephen Peckham, Ana Maria Nogales Vasconcelos

**Affiliations:** 1 Programme of Post-Graduation in Development, Society and International Cooperation, University of Brasilia, Campus Universitário Darcy Ribeiro – UnB, Pavilhão Multiuso I – PMU I, Bloco C 1º andar, Asa Norte, Brasília-DF, Brazil; 2 Centre for Health Services Studies, University of Kent, George Allen Wing, Canterbury CT2 7NF, United Kingdom

**Keywords:** health policy, intersectoral actions, equity, health system strengthening, primary care

The COVID-19 pandemic exacerbated long-standing policy knowledge [1]—and the already historical fact [2]—that health equity issues for the vulnerable population consist of a major challenge in primary healthcare policy and therefore need to be integrated into daily care and management strategies to address people’s social conditions or necessities [3–5].

## Consensus, Questions and Gaps

To improve intersectoral actions and equity in primary healthcare, it is generally agreed that there is a need to better understand the interconnectedness of policymaking and implementation and how to apply multiple analytical approaches. Policymaking and implementation processes need to be better understood in an integrated or interrelated way, considering distinct policy levels (national/state/district, local) and stakeholders involved in the process (policy managers, frontline workers, civil society/community), seeking to see how they can effectively affect both performance and equity/SDH [2–6]. In addition, applying complex or multidisciplinary research/policy models, frameworks or tools is essential to better understand factors that could lead to the improvement of intersectionality and equity in primary health [7, 8].

This raises two important questions for analysts and policymakers:

- How can we more effectively understand and strengthen the process and effects of collaborative intersectoral actions or programs in PHC so as to improve the levels of health equity?
- How should policy-relevant factors and pre-requisites be realistically and seriously considered and employed as contextual evidence in research and policy?

While these questions reflect research and policy-relevant knowledge gaps or challenging factors in low- and middle-income countries (LMIC), they can also be applied to high-income countries (HIC).

### Challenging dimensions and factors

Research evidence, experts and international agencies have recommended different ways to develop stronger collaborative policymaking and implementation processes targeting intersectoral actions and health equity for the vulnerable population [4–6, 9–11].

This would involve the collaboration and co-production between sectors/systems that have traditionally been structured and/or have worked separately. While often leading to collaboration in some initiatives, this has lacked developing institutional mechanisms that could strengthen their collaborations. Stronger implementation processes that promote diverse types of stakeholder engagement and better coordination are also needed [4, 5, 10]. This entails, for example, the construction of policy drivers or mechanisms that can foster community participation and civil society organisations/movements participation, the strengthening of multi-stakeholders’ networks and the generation of more comprehensive and multisectoral forms of evaluation or analysis. Specific implementation areas should be prioritised for better equity, such as delivery and adaptation, monitoring of selected markers, policy dissemination and capacity building, for example [10].

In addition, research and policy initiatives could improve connections between the policy process and capacities needed to promote inter/multisectoral actions addressing pro-equity issues or SDH dimensions. Additionally, initiatives need to promote connections between the policy process and outcomes in terms of policy success and/or system strengthening (services delivery, leadership and workforce). There is also a need to employ new or revised analytical framework(s), policy tools and forms of monitoring, considering cross-sector interactions, connecting concepts from different fields, such as policy and health system research, social care and public policy, as examples [8, 9].

In all these aspects, stronger political commitments will be essential to guarantee more integrated and collaborative forms of formulation, implementation and evaluation [12]. Such approaches will also require addressing current insufficient or lack of funding for intersectoral actions, complexity in aligning priorities between sectors, resistance to changing the ways that stakeholders are engaged, the predominance of policy sectors’ specialised or specific paradigms and barriers to developing capacity-building collaborative efforts.

## The Special Issue

This issue contributes to advancing our understanding of how pro-equity/SDH issues have been formulated and/or implemented, revealing diverse intersectoral actions or being integrated into different PHC strategies/actions, affecting—in various ways—the strengthening of primary healthcare policy around the world. This collection of papers presents three case studies of countries that have been developing multisectoral actions in health. One paper is based on an HIC (Canada) and the two others are focused on an LMIC (India). Papers bring different approaches and aspects on how to improve intersectoral or multisectoral actions to improve and/or strengthen health equity.

Jackson et al. [13] employ a participatory governance approach to study intersectoral collaboration between the community and public sectors while supporting diverse types of immigrants during the COVID-19 pandemic in Toronto. The development of thematic analysis shows that intersectoral actions can bring responsive services for vulnerable immigrants during a pandemic. Authors also emphasise the need to build bottom-up institutional mechanisms to further strengthen collaborative actions during a non-pandemic period.

Desai et al. [14] study the implementation of ‘Rescue Mission’, a policy created to strengthen implementation capability and leadership to improve maternal health. The study shows how diverse forms of participation and interaction at different governance levels enabled the development of technical capabilities and leadership that influenced the institutionalisation of multisectoral collaboration in maternal health. Meghalaya’s experience can be seen as an example of how capabilities and leadership should be employed to enhance collaboration in health.

Nambiar et al. [15] collected new qualitative data from different types of stakeholders—community leaders, healthcare professionals, public health officials and elected members of Local Self-Government (LSG) bodies—to show how local representatives can impact multisectoral actions before and during COVID-19. The results demonstrate that LSG and its members managed to develop multiple roles—as coordinators, gatekeepers and team members, for instance—in plural and flexible ways, taking into account the contexts at the local levels. The key role played by LSG and its members brings policy-relevant lessons on how to foster successful coordination in multisector actions in health.
